# Dose‐escalation strategies which use subgroup information

**DOI:** 10.1002/pst.1860

**Published:** 2018-06-13

**Authors:** Amy Cotterill, Thomas Jaki

**Affiliations:** ^1^ Cancer Research UK Clinical Trials Unit, Institute of Cancer and Genomic Sciences University of Birmingham Birmingham UK; ^2^ Medical and Pharmaceutical Statistics Research Unit, Department of Mathematics and Statistics Lancaster University Lancaster UK

**Keywords:** dose‐escalation, subgroup effect, Bayesian model‐based method, spike and slab

## Abstract

Dose‐escalation trials commonly assume a homogeneous trial population to identify a single recommended dose of the experimental treatment for use in future trials. Wrongly assuming a homogeneous population can lead to a diluted treatment effect. Equally, exclusion of a subgroup that could in fact benefit from the treatment can cause a beneficial treatment effect to be missed. Accounting for a potential subgroup effect (ie, difference in reaction to the treatment between subgroups) in dose‐escalation can increase the chance of finding the treatment to be efficacious in a larger patient population.

A standard Bayesian model‐based method of dose‐escalation is extended to account for a subgroup effect by including covariates for subgroup membership in the dose‐toxicity model. A stratified design performs well but uses available data inefficiently and makes no inferences concerning presence of a subgroup effect. A hypothesis test could potentially rectify this problem but the small sample sizes result in a low‐powered test. As an alternative, the use of spike and slab priors for variable selection is proposed. This method continually assesses the presence of a subgroup effect, enabling efficient use of the available trial data throughout escalation and in identifying the recommended dose(s). A simulation study, based on real trial data, was conducted and this design was found to be both promising and feasible.

## 1 INTRODUCTION

The aim of a dose‐escalation trial is to identify the recommended dose of an experimental treatment to be used in later phase trials investigating the treatment's efficacy. To maximise the treatment's chance of success in efficacy trials, it is important that the recommended dose is optimal for the patient population. Despite this, time restrictions mean that selection of the recommended dose is often based purely on toxicity data which are available relatively soon after treatment. The toxicity data upon which decisions are based is usually a binary indicator of whether a patient experienced a dose‐limiting toxicity (DLT) in their first cycle of treatment.

A common assumption in dose‐escalation trials is that toxicity increases monotonically with dose of the treatment. Since the recommended dose is chosen based only on toxicity data, an implicit assumption is that increasing toxicity leads to increased efficacy of the treatment. Using a Bayesian model‐based design for dose‐escalation, the optimal dose can be referred to as the TD100*θ*.^1^ That is, the dose of treatment with probability *θ* of causing a dose‐limiting toxicity in a patient within their first cycle of treatment. Bayesian model‐based designs require a model to be assumed for the dose‐toxicity relationship. These designs can use available trial data and prior knowledge to advise escalation and estimate the TD100*θ*.

In standard dose‐escalation trials, the trial population is assumed to be homogeneous^2^ and a single TD100*θ* is identified for the entire population. However, in a general patient population this is unlikely to be the case. Variability between subgroups of patients in a population can lead to differences in tolerance or efficacy of the treatment. Consequently, the benefit‐risk ratio of the treatment is impacted for subgroup members. When there is notable variability between subgroups of a population, we refer to the presence of a subgroup effect. Often, the underlying cause of variability is unknown but there can be visible or measurable indicators, referred to as biomarkers, which can be used as intermediate markers of subgroup membership. Examples include ethnicity, pretreatment, or a genetic mutation. For example, presence of a KRAS mutation in patients with non‐small cell lung cancer indicates lower survival when treated with Erlotinib and chemotherapy, than is usual for patients without the mutation.^3^

The limited number of patients available for treatment in dose‐escalation trials makes reliable in‐trial identification of relevant biomarkers unrealistic. Instead, cases where historical information is used to predefine potential biomarkers of interest are considered. For example, historical trials of the same treatment in another application, or of a treatment with similar action being tested in the same application, can be used to identify a biomarker of interest.

Currently, historical data on potential subgroup effects is largely used in the specification of trial inclusion criteria. These can be used to reduce the variability in the trial population to justify an assumption of a homogeneous trial population. In doing this, the population to whom the treatment could be made available is restricted. There is also a risk of excluding patients who could in fact benefit from the treatment. This was the case for Cetuximab which was initially tested in a restricted population of patients with colorectal cancer. It was later noticed that patients excluded from the original trial could in fact benefit from the treatment.^4^ As a consequence, further trials had to be conducted in the additional patient group.

On the other hand, inclusion of a subgroup (in the trial population) in which the treatment is inefficacious could mask a treatment effect in the remaining population. Gefitinib for the treatment of non‐small cell lung cancer is an example where this was the case. On further investigation, the subgroup effect was identified and a reduced population who could benefit from Gefitinib was found.^4^ In both the Cetuximab and Gefitinib examples, the error was highlighted and adjusted for. Unfortunately, there are potentially many similar cases for which the error has not been realised. In addition, more efficient trials, which used fewer resources, could have been implemented if a potential subgroup effect had been accounted for at the initial design stage of these trials.

It is becoming more common for potential subgroup effects to be considered in phases II and III trials (aside from in exploratory analyses). In these so called enrichment trials, subgroup effects are investigated to identify a subgroup of the population who appear most likely to benefit from the treatment (see Temple^5^ for a short overview of such designs). This can lead to exclusion of a subgroup of the patient population from the trial. In such a case, the dose used in the trial was selected based on patients from the initial population and may therefore be suboptimal for the final population. In addition, administering different doses of the treatment between subgroups might suffice, removing the need to completely exclude subgroups from the trial. So, ideally, a TD100*θ* would be estimated in each subgroup, when this is necessary due to presence of a subgroup effect. This could increase the chance of finding the treatment to be efficacious in a larger patient population and is a step towards patient‐specific dosing.

In Section 1.1, a description of a standard Bayesian model‐based method of dose‐escalation is given and the general notation used in the remainder of the paper is introduced. This continues into a brief review of alternative model‐based dose‐escalation designs. In Section 1.2, current methods of accounting for a subgroup effect in clinical trials are discussed. The standard dose‐escalation trial design described in Section 1.1 is used as the underlying design for the proposed methods of accounting for a potential subgroup effect in dose‐escalation. The proposed methods are presented in Section 2 and compared through a simulation study in Section 3. The paper concludes with a discussion of the methods, their limitations and possible extensions in Section 4.

### 1.1 A standard Bayesian model‐based method of dose‐escalation

Bayesian model‐based designs enable available prior and trial information to be used in dose‐escalation decisions. Using all of this available information in dose‐escalation makes escalation decisions more efficient and also safer for patients involved in the trial. The approach of Whitehead & Williamson^1^ is a standard Bayesian model‐based method of dose‐escalation which assumes a homogeneous trial population. Their method is described here and is the design underlying the methods presented in Sections 2.1 and 2.2 for accounting for a potential subgroup effect, as well as being used as the baseline for comparison of the methods.

Dose set *d* of the experimental treatment is to be made available for administration to patients in the dose‐escalation trial. In reality, escalation using a model‐based design is not constrained to this dose set but this is required for the purpose of simulation. Define the dose of treatment administered to a patient as *x* ∈ *d*, and *d* ^∗^ as some fixed reference dose used to standardise dose in the dose‐toxicity model. The probability that a patient experiences a DLT given dose *x* of the experimental treatment, *π*(*x*), is of interest. Specifically, the value of *x* for which *π* (*x*) = *θ*. Escalation under the standard design, assuming a homogeneous trial population, proceeds as follows:

1. Model the dose‐toxicity relationship in the entire population by
  (1) $$\log\left\{\frac{\pi (d)}{1-\pi (d)}\right\}=\beta_{0}+\beta_{1}\log\left(\frac{x}{d^{*}}+1\right)\;\text{where}\;\pi (d)=P(\text{DLT}|d).$$
  The transformed, standardised dose *x*/*d* ^∗^+1 is considered in the assumed dose‐toxicity model to aid interpretation of the model parameters; *β*_0_ is the odds of toxicity at a zero dose of the treatment.
2. Set a prior on the model parameters: This is achieved by specifying pseudo‐data relating to a prior proportion of DLTs occurring at two “prior” doses. The prior data are weighted to total, say 1/10th, of the planned sample size of the trial. Incorporating the pseudo‐data into the dose‐toxicity model in the same way as trial data effectively induces beta priors on the probability of toxicity at the two doses.^6^ The prior proportion of DLTs at the two prior doses can be elicited from clinical experts (as described in Whitehead and Williamson,^1^ for example). Alternatively, the prior can be selected to control the operating characteristics of dose‐escalation. For example, specifying
  - the desired start dose for the trial as the lower of the two prior doses with prior proportion of DLTs at this dose equal to *θ*; and
  - a dose at the top of the planned dose range as the other prior dose with prior proportion of DLTs at this dose selected to control the rate of escalation under some likely trial scenarios.
3. Allocate patients the dose (from set *d*) which, based on the prior and available trial data at their time of recruitment into the trial:
  - maximises the patient gain, $\frac{1}{{\left\{\hat{\pi }(d)-\theta \right\}}^{2}}$,
  - within doses which satisfy the safety criterion, $\hat{\pi }(d)<\delta$,
  for unacceptable level of toxicity *δ* and $\hat{\pi }(d)=1/[1+e^{-\{{\hat{\beta }}_{0}+{\hat{\beta }}_{1}\log(x/d^{*}+1)\}}]$ where ${\hat{\beta }}_{0}$ and ${\hat{\beta }}_{1}$ are the modal a posteriori (MAP) estimates of the model parameters. When prior knowledge is incorporated into the dose‐toxicity model as pseudo‐data, the MAP estimates are equivalent to the maximum likelihood estimates of the parameters and so standard software can be used without the need for Markov Chain Monte Carlo (MCMC).
4. Stop escalation:
  - For safety if, at any point in the trial, no available doses satisfy the safety criterion, no recommended dose is declared. Or,
  - Once a maximum number of patients have been treated in the trial, the recommended dose is declared as the estimated TD100*θ* for the entire population based on data collected in the trial (ie, not including prior pseudo‐data). That is, the dose which maximises the patient gain and satisfies the safety criterion (based on the two‐parameter dose‐toxicity model of Equation 1), from the range of available doses which are less than or equal to the maximum dose administered during the trial.

Other authors, such as Neuenschwander et al,^7^ have assumed the same two‐parameter dose‐toxicity model for dose‐escalation. Their approach differs in specification of escalation rules for the trial (Step 3). Whitehead and Williamson^1^ themselves suggest alternative escalation rules to that described here but the patient gain has been chosen as it is the most ethical option. Addition of the safety constraints in a similar manner to Babb et al^8^ controls the rate of escalation, improving the safety of the trial for the patients involved.

Alternative dose‐toxicity models have been suggested; the continual reassessment method (CRM) of O'Quigley et al^9^ uses a one‐parameter power model which accurately estimates the TD100*θ* but does not effectively model the entire dose‐toxicity relationship. Goodman et al,^10^ among others, have proposed modifications on the CRM to reduce the aggressiveness of escalation. Other Bayesian model‐based designs have been proposed which aim to optimise escalation, although these are often considered unethical as they do not account for the needs of patients.^11^, ^12^ Reviews of dose‐toxicity models and available methods of dose‐escalation are provided in Rosenberger and Haines^2^ and Jaki et al. 13

Most Bayesian model‐based dose‐escalation trial designs have the same foundations and so the methods presented in this paper could be adjusted for the use of an alternative dose‐toxicity model or escalation rules. A two‐parameter model was selected in this case as being more suitable than a one‐parameter model for comparison of the dose‐toxicity relationship between subgroups. This is because, although the subgroup effect may not affect the recommended dose itself, differences in the shape of the dose‐toxicity curves between subgroups may indicate a subgroup effect that will be more obvious in later trials with different endpoints. As with any Bayesian trial design, simulation should be conducted prior to implementation. Simulations should consider a range of potential data scenarios as well as reasonable prior settings.

### 1.2 Current methods of accounting for subgroup information in clinical trials

The most straight‐forward way to account for a subgroup effect in dose‐escalation is to stratify by subgroup membership and conduct independent dose‐escalation in each subgroup. This has been done in practice (eg, Nicholson et al^14^) but is inefficient (in its use of information for identifying a dose for escalation and estimating the TD100*θ*), especially if there is in fact no underlying subgroup effect. Wijesinha et al^15^ and O'Quigley et al^16^ propose using additional terms in the dose‐escalation model to account for subgroup membership. In this way, some information is shared between subgroups during escalation. Babb et al^17^ use a similar method but consider a continuous biomarker; their design is demonstrated in Cheng et al.^18^

Neuenschwander et al^19^ present an approach for subgroup‐based escalation in a setting where pooling of data is deemed inappropriate but sharing of information between subgroups is desirable. This setting is different to the one considered in this paper where data come from an overall population with a suspected subgroup effect. Guo and Yuan^20^ present a two‐stage design with data pooled in the first stage. In the second stage, toxicity and efficacy data are used together with covariate information to recommend patient‐specific doses. Novel bridging methods have been developed in relation to dose‐finding studies (eg, Liu et al^21^ and O'Quigley and Iasonos^22^). These methods aim to address a related but different question to subgroup‐based escalation.

In current practice, it is more common for a subgroup effect to be investigated in later phase trials. Such designs use hypothesis testing at an interim point in the trial to identify subgroup(s) of the population that react favourably to treatment and, hence, are felt worth pursuing for further investigations of the experimental treatment.^4^, ^23^, ^24^

## 2 PROPOSED METHODS OF ACCOUNTING FOR SUBGROUP INFORMATION IN DOSE‐ESCALATION

When the trial population is truly homogeneous, a standard method of dose‐escalation (such as that of Whitehead and Williamson^1^ described in Section 1.1), which does not account for a potential subgroup effect, is suitable. However, this design is not appropriate when there is uncertainty around the assumption of a homogeneous population. We compare the standard design (which assumes a homogeneous population) to two alternative methods of dose‐escalation which account for subgroup membership throughout escalation. The first of these (presented as Method 1 in Section 2.1) extends the dose‐toxicity model to include terms for subgroup membership. The dose‐toxicity method used is effectively a different parameterisation of that presented by O'Quigley et al.^16^ The second method (presented as Method 2 in Section 2.2) is the novel method presented in this manuscript.

Say that patients entering the trial can be reliably classified as being in one of two distinct, clearly identifiable subgroups based on the presence or absence of a predefined biomarker. The treatment is expected to be more toxic in biomarker positive patients than in the remaining biomarker negative patients. Let ${𝕝}_{+}$ be an indicator of subgroup membership which is equal to 1 for a biomarker positive patient and 0 for a biomarker negative patient.

### 2.1 Method 1: Include terms for subgroup membership

In this method, the standard two‐parameter dose‐toxicity model from Equation 1 is extended to include terms for subgroup membership. This enables escalation decisions to be made which account for subgroup membership. Hence, making the dose administered to patients better suited to them. A consequence of allowing escalation to differ between subgroups is that the safety stopping criterion can come into play for one or both subgroups. Escalation under this method proceeds as follows:

1. Model the dose‐toxicity relationship using the four‐parameter logistic model:
  (2) $$\begin{array}{ccc} \log\left\{\frac{\pi (d)}{1-\pi (d)}\right\} & =\beta_{0}+\beta_{1}\log\left(\frac{x}{d^{*}}+1\right)+{𝕝}_{+}\left\{\beta_{2}+\beta_{3}\log\left(\frac{x}{d^{*}}+1\right)\right\}, & \\ \;\text{where}\;\pi (d) & =P(\text{DLT}|d,{𝕝}_{+}). \end{array}$$
  If historical evidence of a subgroup effect led to strong belief of its impact on either the intercept or slope parameter of the dose‐toxicity model, then one of the additional terms could be removed and the resulting three‐parameter model used in place of the four‐parameter model. However, with a lack of information on the expected impact of the subgroup effect on the dose‐toxicity relationship, the four‐parameter dose‐toxicity model is able to capture potential variability in both parameters.
2. Set a prior on the model parameters: This can be achieved in a similar manner to that for the standard design by specifying pseudo‐data on two prior doses for the biomarker positive subgroup and two prior doses for the biomarker negative subgroup. The pseudo‐data for each subgroup is weighted to, say 1/10th, of the planned sample size in that subgroup.
3. Allocate patients the dose (from set *d*) which, based on their subgroup membership, the prior and available trial data at their time of recruitment into the trial:
  - maximises the patient gain, $\frac{1}{{\left\{\hat{\pi }(d)-\theta \right\}}^{2}}$,
  - within doses which satisfy the safety criterion, $\hat{\pi }(d)<\delta$,
  for unacceptable level of toxicity *δ* and for MAP estimates of the model parameters ${\hat{\beta }}_{0}$, ${\hat{\beta }}_{1}$, ${\hat{\beta }}_{2}$, and ${\hat{\beta }}_{3}$ with $\hat{\pi }(d)=1/(1+e^{-[{\hat{\beta }}_{0}+{\hat{\beta }}_{1}\log(x/d^{*}+1)+I_{+}\{{\hat{\beta }}_{2}+{\hat{\beta }}_{3}\log(x/d^{*}+1)\}]})$.
4. Stop escalation:
  - For safety in a subgroup if, at any point in the trial no available doses satisfy the safety criterion for that subgroup; no recommended dose is declared in that subgroup. Escalation continues in the other subgroup using the two‐parameter model of Equation 1 fitted to data from patients in the remaining subgroup only.
  - Once a maximum number of patients have been treated in the trial:
    ∘ If one subgroup stopped for safety, the recommended dose is declared in the remaining subgroup as the estimated TD100*θ* based on data collected in the trial (ie, not including prior pseudo‐data). That is, the dose which maximises the patient gain and satisfies the safety criterion (based on the two‐parameter dose‐toxicity model of Equation 1 fitted to the data from patients in that subgroup only), from the range of available doses which are less than or equal to the maximum dose administered to patients in the respective subgroup during the trial.
    ∘ If neither subgroup stopped for safety, a recommended dose is declared in each subgroup as the estimated TD100*θ* based on data collected in the trial (ie, not including prior pseudo‐data). That is, the dose which maximises the patient gain and satisfies the safety criterion (based on the four‐parameter dose‐toxicity model of Equation 2) from the range of available doses which are less than or equal to the maximum dose administered to patients in the respective subgroup during the trial.

By including covariates for subgroup membership in the dose‐toxicity model, this method of dose‐escalation enables recommended doses to be subgroup specific. A TD100*θ* is estimated in each subgroup (unless one or both subgroups stop for safety). When these recommendations differ between subgroups, then it is expected that a significant subgroup effect has been observed. When the recommendations are the same between subgroups, this could be down to there truly being no significant subgroup effect. On the other hand, it could be a result of the discrete dose set or insufficient sample size in the trial to detect a difference.

Although the dose recommended for treatment of members of both subgroups in future trials might be the same, the subgroup effect may become clear in the longer‐term, or when efficacy outcomes are investigated. Even exploratory inferences could be beneficial to obtain an idea of whether a subgroup effect was observed in the dose‐escalation trial, aiding design of future trials. The use of a hypothesis test to achieve this was considered but found to be low‐powered. In addition, there is no consideration in this method that there may be no subgroup effect. If this is in fact the case, then this method uses data inefficiently throughout escalation and in identifying the final recommended doses.

### 2.2 Method 2: Fully Bayesian method using spike and slab priors for variable selection

This method is based on the four‐parameter dose‐toxicity model given in Equation 2. In Method 1, the four‐parameter dose‐toxicity model was used throughout escalation and no inference over presence or absence of a subgroup effect was made. It would be more efficient to decide at each escalation step, based on data available at that time, whether the two‐ or four‐parameter dose‐toxicity model is more suitable. Ideally, the entire dose‐toxicity curve would be considered in this test; the frequentist alternative which can achieve this using hypothesis testing is too low‐powered to be practical.

**Figure 1:**
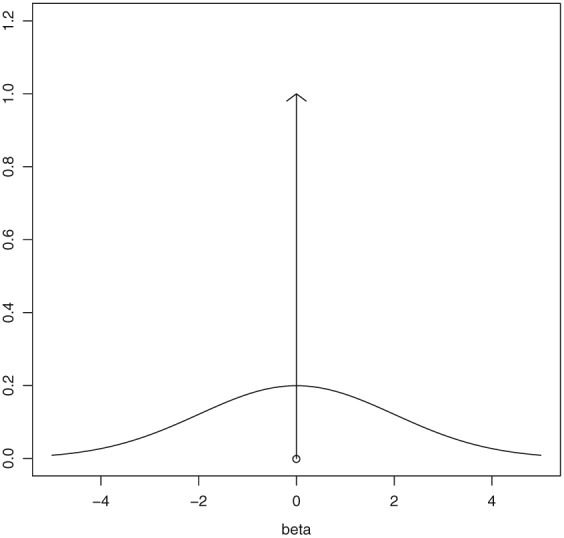
Example of a mixture prior on β composed of a normal slab and Dirac delta function spike

The Bayesian alternative that we propose overcomes these problems to some extent by using spike and slab priors on the model terms for subgroup membership (*β*_2_ and *β*_3_ in Equation 2). A spike and slab prior is effectively a two‐component mixture prior. One component is usually a normal prior with high variance which makes up the “slab” part of the prior. The other part is the “spike” component which is selected as a distribution which has a large mass at zero. We choose to use a Dirac delta function, *δ*_0_ (a point mass at zero), which results in a sparsity inducing spike and slab mixture prior. Figure 1 gives an example of a potential mixture prior on *β* composed of a normal slab and Dirac delta function spike. The result of using these spike and slab priors is that a positive probability is placed on the probability of the term being equal to zero. Based upon this, spike and slab priors can be used in choosing the model.

Take *γ*_2_ to be a latent indicator function which indicates inclusion (when equal to 1, and is zero otherwise) of the variable *β*_2_ in the dose‐toxicity model. The resulting spike and slab prior on *β*_2_ can be written as

$$\beta_{2}|\gamma_{2}∼\gamma_{2}N(0,\sigma_{2}^{2})+(1-\gamma_{2})\delta_{0}.$$

The decision over whether *β*_2_ is required in the model, based on available data, can be based on its probability of inclusion in the model, *w*_2_. This can be estimated by placing a Bernoulli prior on *γ*_2_ such that

$$P(\gamma_{2})=w_{2}^{\gamma_{2}}{\left(1-w_{2}\right)}^{\left(1-\gamma_{2}\right)}.$$

Similarly, we can consider a latent indicator function *γ*_3_ and probability of inclusion *w*_3_ on *β*_3_. Assume that *w*_2_ is independent of *w*_3_ and, as such, a prior setting of *w*_2_ = *w*_3_ = 0.5 implies a prior belief that one of the two predictors for subgroup effect is significant in the model (see Chapter 10 of Do et al^25^). If instead *w*_2_ or *w*_3_ were set equal to 1, then the corresponding term would be forced into the model with a normal prior (the slab component of the prior corresponding to that term) placed on it. This is effectively done for *β*_0_ and *β*_1_ which are required in the dose‐toxicity model.

A range of algorithms exist for implementing Bayesian model selection using spike and slab priors in the linear regression setting (eg, George et al^26^, Ishwaran & Rao^27^ and Scheipl^28^). Authors such as Wagner and Duller^29^ and Tüchler^30^ have extended these methods to the logistic regression setting. The applications of Bayesian variable selection for logistic regression models is wide‐ranging; Wagner and Duller 29 aim to identify relevant risk factors for bleeding while Genkin et al^31^ is concerned with text categorisation. Methods which deal with multivariate regression and ANOVA are also available (eg, Carvalho et al^32^) which have application in selection of variables relating to gene expression.

When spike and slab priors are used, there is a form of in‐built decision‐making process over whether the additional terms are required in the model. Once the relevant variables have been identified, the selected model is fitted to the data and escalation decisions can be made based upon this. Escalation decisions now occur in two stages: choosing the model and model fitting. An alternative to having these two steps in escalation would be to use posterior modal parameter estimates from the spike and slab model in selecting the dose for escalation. The parameter estimates obtained from this alternative method would be shrunk towards zero, hence not reflecting presence of a subgroup effect to its full extent. It is for this reason that we chose not to use it.

Escalation under this method proceeds as follows:

1. Model the dose‐toxicity relationship using the four‐parameter logistic model:
  $$\begin{array}{ccc} \log\left\{\frac{\pi (d)}{1-\pi (d)}\right\} & =\beta_{0}+\beta_{1}\log\left(\frac{x}{d^{*}}+1\right)+{𝕝}_{+}\left\{\beta_{2}+\beta_{3}\log\left(\frac{x}{d^{*}}+1\right)\right\}, & \\ \text{where}\;\pi (d) & =P(\text{DLT}|d,{𝕝}_{+}). \end{array}$$
  The terms *β*_0_ and *β*_1_ will always be included in the model used for escalation. However, spike and slab priors are specified on *β*_2_ and *β*_3_ and so one or both of these terms could be set to zero in the model for escalation.
2. Set a prior on the model parameters: Pseudo‐data of the same form used in Method 1 is used to define the priors.
  Model fitting: Fit pseudo‐data to the four‐parameter logistic regression model of Equation 2. The resulting coefficient estimates are used to derive the slab component of the priors on the four parameters of the dose‐toxicity model. The prior weight of the spike component for each parameter is also specified; this will be zero for *β*_0_ and *β*_1_ and greater than zero for the terms for subgroup membership, *β*_2_ and *β*_3_. For the prior, we choose to include *β*_2_ and *β*_3_ in the model, regardless of their value (ie, whether it is greater than or less than the prior inclusion probability).
3. Escalation follows the two‐step process:
  Choosing the model: Fit the spike and slab model using MCMC. After removing burn‐in iterations, find *w*_2_ and *w*_3_ (the probability that each term was included in the dose‐toxicity model which is always 1 for *β*_0_ and *β*_1_ but varies for *β*_2_ and *β*_3_). If the inclusion probability of the parameter is greater than some prespecified boundary, then that term will be non‐zero in the fitted model. Otherwise, it is equal to zero for this model update.
  Model fitting: Allocate patients the dose which, based on their subgroup membership (if relevant), the prior and available trial data at their time of recruitment into the trial:
    - maximises the patient gain, $\frac{1}{{\left\{\hat{\pi }(d)-\theta \right\}}^{2}}$,
    - within doses which satisfy the safety criterion, $\hat{\pi }(d)<\delta$,
  for unacceptable level of toxicity *δ* and $\hat{\pi }(d)=1/[1+e^{-\{{\hat{\beta }}_{0}+{\hat{\beta }}_{1}\log(x/d^{*}+1)+y\}}]$, where *y* is the term(s) for subgroup membership identified for inclusion in the model during variable selection. The estimates ${\hat{\beta }}_{0}$, ${\hat{\beta }}_{1}$, and potentially ${\hat{\beta }}_{2}$ and/or ${\hat{\beta }}_{3}$, are the MAP estimates of the dose‐toxicity model parameters.
4. Stop escalation:
  - For safety in a subgroup if, at any point in the trial, no available doses satisfy the safety criterion for that subgroup; no recommended dose is declared in that subgroup. Escalation continues in the other subgroup using the two‐parameter dose‐toxicity model of Equation 2 fitted to data from patients in that subgroup only. Or,
  - Once a maximum number of patients have been treated in the trial:
    ∘ If one subgroup stopped for safety, the recommended dose is declared in the remaining subgroup as the estimated TD100*θ* based on data collected in the trial (ie, not including prior pseudo‐data). That is, the dose which maximises the patient gain and satisfies the safety criterion (based on the two‐parameter dose‐toxicity model of Equation 1 fitted to the data from patients in that subgroup only), from the range of available doses which are less than or equal to the maximum dose administered to patients in the respective subgroup during the trial.
    ∘ If neither subgroup stopped for safety: Conduct the variable selection step,
      * If both *β*_2_ and *β*_3_ are equal to zero, the data are pooled and a single recommended dose is declared for the entire population as the estimated TD100*θ* based on data collected in the trial (ie, not including prior pseudo‐data). That is, the dose which maximises the patient gain and satisfies the safety criterion (based on the two‐parameter dose‐toxicity model of Equation 1), from the range of available doses which are less than or equal to the maximum dose administered during the trial.
      * If *β*_2_ and/or *β*_3_ is non‐zero: As in Method 1, a recommended dose is declared in each subgroup as the estimated TD100*θ* based on data collected in the trial (ie, not including prior pseudo‐data). That is, the dose which maximises the patient gain and satisfies the safety criterion (based on the four‐parameter dose‐toxicity model of Equation 2), from the range of available doses which are less than or equal to the maximum dose administered to patients in the respective subgroup during the trial.

The overall set‐up of this method is relatively similar to the previous methods. However, before model fitting can occur in Step 3, the model must be chosen (and a relevant prior specified). The use of spike and slab priors mean that the model used in choosing the model is not conjugate and so MCMC is required, making Method 2 more computationally complex than the previous methods.

The use of spike and slab priors on the terms for subgroup membership enables escalation decisions to be founded on the most relevant model based on all data available at that stage of the trial. This makes escalation more efficient and so can be beneficial for patients. In addition, by considering whether each variable should be included in the model, the entire dose‐toxicity curve is compared between subgroups. This is in comparison to looking merely at point estimates of the dose recommended in each subgroup, as done in Method 1.

In this method, there is no formal test of whether a subgroup effect was observed and so the decision over the presence or absence of a subgroup effect is exploratory. This exploratory conclusion, together with historical information and clinical expertise on the expected subgroup effect, may be suitable to decide whether a subgroup effect should be accounted for in later phase trials. Alternatively, a hypothesis test could be conducted on the final trial data with no adverse effect on escalation, although this has the aforementioned issues.

## 3 SIMULATION STUDY

Data from the single‐agent paediatric dose‐escalation trial reported by Nicholson et al^14^ was used as the basis for the simulation study presented in this section. In the reported trial, Nicholson et al used stratification to account for a potential subgroup effect and escalation proceeded in each subgroup under an “up and down” design (see Storer^33^ for an example of such a design). In this trial, biomarker positive patients had experienced a specific line of prior treatment which the biomarker negative patients had not. The decision to stratify by this prior treatment came from evidence obtained in adult trials of the treatment.

The data obtained in the trial are given in Table 1, both by subgroup membership and as the pooled data. Based upon the algorithmic design and definition of the recommended dose specified by Nicholson et al, the maximum tolerated doses were identified as 215 and 180 mg/m^2^ in the biomarker negative and biomarker positive subgroups, respectively. Now, had the two‐parameter dose‐toxicity model in Equation 1 been used during the course of the trial, the data obtained and resulting recommended doses are likely to have been different. These trial data are used in this manuscript as a basis for the simulation study; no attempt is made to re‐evaluate the outcomes of the trial reported by Nicholson et al. The parameter estimates resulting from fitting the dose‐toxicity model in Equation 1 to the data give a TD16 in the biomarker positive subgroup that is very similar to that under the algorithmic design at 181 mg/m^2^. However, in the biomarker negative subgroup, the TD16 is 244 mg/m^2^ under the model‐based approach. It is the TD16 that we aim to identify in the simulation study in the remainder of this section.

**Table 1:** Toxicity data observed in the dose‐escalation trial reported in Nicholson et al,^14^ given by subgroup membership and as the pooled data. Also given is the recommended dose declared from the trial based on escalation by an algorithmic design in each subgroup, and the TD16 (given a continuous range of doses) based on fitting the dose‐toxicity model in Equation 1 to the data

	Number of DLTs Observed by Dose (mg/m^2^)							Recommended Dose (mg/m^2^) Based on	
	100	150	180	215	245	260	Total	Algorithmic Design	Model‐Fit to Data
${𝕝}_{+}=0$ subgroup	0/5	0/4	0/4	0/6	2/7	1/1	3/27	215	244
${𝕝}_{+}=1$ subgroup	1/6	0/4	0/8	2/4	‐	‐	3/22	180	181
Pooled data	1/11	0/8	0/12	2/10	2/7	1/1	6/49	‐	206

This simulation study is presented to illustrate the dose‐escalation methods described in Section 2. We compare the methods of dose‐escalation which account for subgroup information to the baseline method; the standard Bayesian model‐based method of dose‐escalation presented in Section 1.1. The simulation setting and scenarios are detailed in Section 3.1. In Section 3.1.1, step‐by‐step implementation (including sections of R code) of the proposed method of dose‐escalation which accounts for subgroup information through use of a spike and slab prior is provided.

### 3.1 Simulation study design

The dose set available for the trial was specified as that used by Nicholson et al,^14^ *d*={100, 150, 180, 215, 245, 260} mg/m^2^. The recommended dose from adult trials was 200 mg/m^2^; this was selected as the reference dose used to standardise doses in the dose‐toxicity model. The starting dose for the trial was taken as the lowest available dose of 100 mg/m^2^ and we specified *θ*=0.16 and set the unacceptable probability of toxicity, for use in the safety criterion, as *δ*=0.35. So, we aimed to identify the dose, from those available, which was less than or equal to the maximum dose administered in the trial and had posterior probability of causing a DLT in a patient closest to 0.16 but less than 0.35.

It is considered that, upon entry to the trial, patients were reliably identified as being either biomarker positive or biomarker negative. Patients were recruited in cohorts of size 2 throughout the trial. Each cohort consisted of one biomarker positive and one biomarker negative patient unless one subgroup stopped escalation early, in which case both patients in the cohort were from the remaining subgroup. The maximum number of patients to be treated in the trial was 60. If neither subgroup stopped escalation early, then this would be made up of 30 patients from each subgroup. In the case of the baseline method, escalation continued until 60 patients had been treated in the trial unless the trial stopped early for safety. Although this might not be realistic, it was used in the simulation study to enable comparison of the methods with a fixed amount of information.

The prior was specified such that it was worth 1/10th of the planned sample size. That is, a total of 6 prior patients consisting of 3 on each subgroup. We specified the same prior data in both subgroups. This was done to aid comparability of the methods but could of course be altered for use in a real trial. After running a range of potential pseudo‐data specifications (details of these are given in Appendix A) the prior data specification selected is presented in Table 2. Under this prior specification, the dose‐toxicity model advises a start dose of 100 mg/m^2^(ie, fitting only the pseudo‐data to the dose‐toxicity model, the escalation rule advises a dose of 100 mg/m^2^ for escalation). In addition, under the scenario of no DLTs, the chosen prior leads to reasonable paced escalation with no skipped doses. Upon observation of a DLT at a low dose, it was felt likely for the model to re‐escalate within the specified maximum trial size. Clearly, these properties differ between the baseline approach and an approach which considers potential subgroup effect. For comparability between methods, our chosen prior is acceptable under both settings.

**Table 2:** Prior pseudo‐data setting used in the simulation study given in terms of the prior proportion of DLTs observed at the lower and higher prior dose with the number of prior patients considered at that dose given in brackets. Pseudo‐data is presented by subgroup (totalling 3 patients worth of data per subgroup) and overall (totalling 6 patients worth of data)

	Prior Pseudo‐data DLT Outcomes by Dose (mg/m^2^)	
	100	260
${𝕝}_{+}=0$ subgroup	1/6 (2)	1/2 (1)
${𝕝}_{+}=1$ subgroup	1/6 (2)	1/2 (1)
Pooled data	1/6 (4)	1 (2)

In the simulation study, toxicity data were generated from the four‐parameter dose‐toxicity model given in Equation 2. The parameter values of *β*_0_ and *β*_1_ used for data generation were the mean estimates obtained from a frequentist model fit to Equation 1 using the pooled trial data (given in Table 1). The parameter values for *β*_2_ and *β*_3_ were varied depending upon the simulated scenario. A “true” probability of DLT refers to the probability of DLT based upon the dose‐toxicity model and parameter values from which data were simulated. Similarly, a “true” recommended dose refers to the dose, from the discrete set available for the trial, which has estimated probability of causing a DLT in a patient closest to the TD16 (from those estimates less than 0.35) based upon the model and parameter values from which data were simulated.

Simulations for all methods were conducted using *R*.^34^ Method 2 required the addition of a model selection step in the escalation procedure compared to the other methods. This step was conducted using the *BoomSpikeSlab* package^35^ which is based on variable selection for logistic regression models as described by Tüchler.^30^ Given that we had no outside information to suggest otherwise, the default settings were used for most parameters required by the functions called from *BoomSpikeSlab*. Running the Markov Chain for 20 000 iterations and removing 5 000 as burn‐in was found to be suitable for convergence. We set the prior inclusion probability for *β*_2_ and *β*_3_ equal to 0.5; this is a relatively non‐informative setting. We specified that a parameter was non‐zero in the fitted model if it had posterior probability of inclusion in the model greater than 0.25. The effect of the prior inclusion probability and probability for inclusion of the terms in the model were investigated. Results of this investigation are given in the sensitivity analysis presented in Appendix B along with investigations into specification of the underlying model.

Results are presented for the following 6 scenarios based on estimates from 1 000 simulated trials under the given scenario and method. The true probabilities of toxicity at each available dose for each of the scenarios are given in Table 3. Further scenarios were run and the results were consistent with those presented here:

1. No subgroup effect: This scenario is included for comparison of the methods when the “true” recommended dose is the same for both subgroups. This could arise when the population is truly homogeneous, or when the biomarker considered in the trial is not the cause of the subgroup effect observed in the trial.
2. A small subgroup effect: Causing only one dose level difference in true recommended doses between subgroups. This scenario is included to investigate the sensitivity of the methods to small differences in tolerance to the treatment between the subgroups.
3. A medium subgroup effect: Causing 2 dose level difference in true recommended doses between subgroups. This scenario, and the next, is included to investigate the sensitivity of the methods to varying degrees of subgroup effect.
4. A medium subgroup effect: Causing 3 dose level difference in true recommended doses between subgroups.
5. A large subgroup effect: No safe dose in the biomarker positive subgroup and a true recommended dose in the biomarker negative subgroup in the middle of the available dose range.
6. No safe dose in either subgroup: This scenario is included to demonstrate the effectiveness of the safety criterion when there are no safe doses in either subgroup.

**Table 3:** Simulated probability of DLT at each dose (in mg/m^2^) under each simulation scenario, given for each subgroup. Grey cells highlight dose‐pairs with probability of causing a DLT in a patient greater than 0.35. The “X” marks the dose with probability of toxicity closest to 0.16, in cases where there is a tolerated dose

	P(DLT|*d*, ${𝕝}_{+}=0$)						P(DLT|*d*, ${𝕝}_{+}=1$)					
Scenario	100	150	180	215	245	260	100	150	180	215	245	260
1	0.02	0.06	0.10	0.18^*X*^	0.28	0.33	0.02	0.06	0.10	0.18^*X*^	0.28	0.33
2	0.02	0.06	0.10	0.18^*X*^	0.28	0.33	0.02	0.08	0.14^*X*^	0.26	0.38	0.45
3	0.02	0.06	0.10	0.18^*X*^	0.28	0.33	0.03	0.13^*X*^	0.24	0.42	0.58	0.65
4	0.02	0.06	0.10	0.18^*X*^	0.28	0.33	0.09^*X*^	0.36	0.60	0.81	0.90	0.93
5	0.02	0.06	0.10	0.18^*X*^	0.28	0.33	0.42	0.90	0.97	0.99	1.00	1.00
6	0.38	0.67	0.79	0.88	0.93	0.94	0.38	0.67	0.79	0.88	0.93	0.94

#### 3.1.1 Implementation in R

The first step in implementing the proposed dose‐escalation method which uses spike and slab priors to account for subgroup information is the same as for any trial; specify the design parameters. These are the doses available for administration in the trial (Doses), the reference dose (Dref), the “target” probability of DLT (*θ*) and an unacceptable probability of DLT (*δ*).

Next, the prior pseudo‐data is specified: subgroup membership, dose, number of patients assumed to experience DLTs, and number without DLTs. The availability of relevant historical data and clinical experience of the trial drug will influence how this prior pseudo‐data is specified. Be this to reflect clinical knowledge, or to control operating characteristics of the trial, or a combination of the two. Simulations are required to evaluate the operating characteristics of the prior to confirm suitability of the selection; graphical and visual methods for calibrating priors have been described.^36^, ^37^ In our case, the prior was chosen to control escalation and was selected to have a weight of 1/10th of the total trial sample size with the same prior assumed for both subgroups.

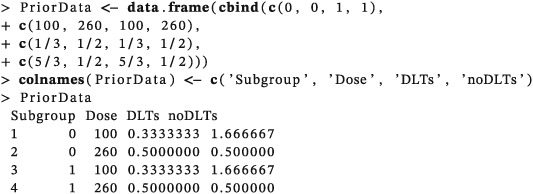

The specified prior implies we have 3 pseudo patients in the biomarker negative subgroup with:

- 2 pseudo patients treated at 100 mg/m^2^ with 1/3 of them having a toxicity and 5/3 having no toxicity.
- 1 pseudo patient treated at 260 mg/m^2^ with 1/2 of them having a toxicity and 1/2 having no toxicity.

Similarly, for the biomarker positive subgroup. Note that fractions of patients are possible for the prior pseudo‐data specification which allows the strength of the prior to be chosen freely.

The model parameters must now be defined. As with the prior specification, there is no definitive method of specifying these parameters and, as they impact on the operating characteristics of the trial, simulation should be conducted to identify suitability parameter values. Malsiner–Walli and Wagner^38^ discuss the specification of spike and slab priors for variable selection. In our case, the prior inclusion probabilities were specified as 1 for *β*_0_ and *β*_1_ (which are always included in the model) and 0.5 for *β*_2_ and *β*_3_. The inclusion bounds for *β*_2_ and *β*_3_ were specified as 0.25.

At this stage, the prior pseudo‐data is used to obtain the spike and slab prior using the function “SpikeSlabPrior” from R package *BoomSpikeSlab*.^35^

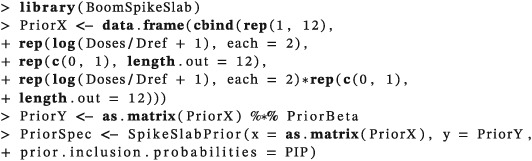

MAP estimates of ${\hat{\beta }}_{0}$, ${\hat{\beta }}_{1}$, ${\hat{\beta }}_{2}$, and ${\hat{\beta }}_{3}$, are obtained using function “glm” and then used to identify the recommended dose for escalation as that which

- maximises the patient gain, $\frac{1}{{\left\{\hat{\pi }(d)-\theta \right\}}^{2}}$,
- within doses which satisfy the safety criterion, $\hat{\pi }(d)<\delta$,

for $\hat{\pi }(d)=1/[1+e^{-\{{\hat{\beta }}_{0}+{\hat{\beta }}_{1}\log(x/d^{*}+1)+y\}}]$ where *y* is the term(s) for subgroup membership identified for inclusion in the model during variable selection.

The prior that we specified leads to a dose of 100 mg/m^2^ being recommended for administration to the first cohort of subjects (whether biomarker positive or biomarker negative). Now, say that the first cohort is composed of two patients, one biomarker positive and one biomarker negative, with a DLT observed in the biomarker positive patient but not in the biomarker negative patient. The data matrix is updated to contain both prior pseudo‐data and observed responses.

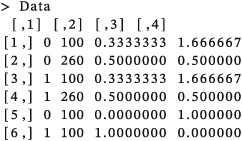

The posterior inclusion probabilities of the model parameters are updated using the function “logit.spike” based on the updated data matrix (Data) and the four‐parameter logistic regression model (Model4para), $\beta_{0}+\beta_{1}\log\left(\frac{x}{d^{*}}+1\right)+{𝕝}_{+}\left\{\beta_{2}+\beta_{3}\log\left(\frac{x}{d^{*}}+1\right)\right\}$.

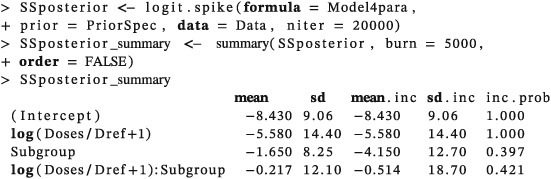

The output shows that the resulting posterior inclusion probabilities for *β*_2_ and *β*_3_ were 0.397 and 0.421 in this case. Both of the posterior inclusion probabilities are greater than the specified inclusion bounds for *β*_2_ and *β*_3_ of 0.25. This means that MAP estimates of the coefficients will be obtained using function “glm” with a model which includes terms with coefficients ${\hat{\beta }}_{0}$, ${\hat{\beta }}_{1}$, ${\hat{\beta }}_{2}$, and ${\hat{\beta }}_{3}$. The dose recommended for the next cohort will therefore depend on the patient's subgroup membership.

If, instead, the inclusion bound had been specified as 0.4 instead of 0.25 for *β*_2_ and *β*_3_. Then, in this case, the posterior inclusion probability for *β*_2_ was less than 0.4 but that for *β*_3_ was greater than 0.4. In this case, the MAP estimates for recommending a dose for escalation would be based on a three‐parameter model: $\beta_{0}+\beta_{1}\log\left(\frac{x}{d^{*}}+1\right)+{𝕝}_{+}\left\{\beta_{2}+\beta_{3}\log\left(\frac{x}{d^{*}}+1\right)\right\}$.

Once data from the next cohort is observed, the posterior inclusion probabilities are found and based on these, the coefficient estimates are updated. Updated estimates are used to recommend the next dose for escalation dependent on the subgroup membership of the patient. This process repeats until the maximum number of subjects have been treated in the trial.

### 3.2 Simulation study results

The standard Bayesian model‐based dose‐escalation trial design described in Section 1.1 (based on the assumption of a homogeneous trial population) is used as the baseline method for comparison of the proposed dose‐escalation methods described in Section 2, which account for a potential subgroup effect. When recommended dose(s) are referred to, these are the frequentist estimates; they are obtained by fitting the relevant logistic regression model to the trial data only (ie, not including prior pseudo‐data). The prior used for the simulation study was selected to control the operating characteristics of the trial; it was not based on real trial data. For this reason, it is not appropriate for the prior data to affect the final outcome of the trial. If, however, the prior was selected based on historical data, then it may be desirable to consider this data in identifying the recommended dose(s) from the trial. Even in such a setting, a frequentist estimate might be used to reduce the subjectivity of decisions made from the dose‐escalation trial that could impact on future trials of the treatment.

From Table 4, it can be seen that in Scenarios 1 to 4, where there was a tolerated dose available for each subgroup, most trials ran to the maximum number of patients with less than 10*%* of trials stopping early for safety in one subgroup. In these scenarios, the average proportion of toxicities observed overall was between 12% and 16*%*. Although the average proportion of toxicities observed was fairly consistent across scenarios in the biomarker negative subgroup (under Methods 1 and 2), that in the biomarker positive subgroup increased as the true subgroup effect increased. This is in part due to the higher toxicity levels of all available doses.

**Table 4:** Average number of patients treated per trial in total and in each subgroup, average proportion of toxicities observed per trial in total and in each subgroup

	Escalation	Average Number Patients			Average Proportion Toxicities		
Scenario	Method	Overall	${𝕝}_{+}=0$	${𝕝}_{+}=1$	Overall	${𝕝}_{+}=0$	${𝕝}_{+}=1$
1	Baseline	59.94	29.97	29.97	0.12	0.12	0.12
	1	58.59	29.45	29.14	0.12	0.14	0.15
	2	58.97	29.49	29.48	0.12	0.14	0.13
2	Baseline	60.00	30.00	30.00	0.12	0.10	0.15
	1	58.79	29.42	29.37	0.13	0.14	0.15
	2	58.96	29.48	29.48	0.13	0.13	0.15
3	Baseline	60.00	30.00	30.00	0.13	0.08	0.19
	1	58.36	29.57	28.80	0.14	0.13	0.18
	2	58.04	29.34	28.71	0.14	0.14	0.19
4	Baseline	59.67	29.84	29.84	0.16	0.05	0.27
	1	56.40	29.36	27.04	0.14	0.14	0.23
	2	56.38	29.45	26.93	0.15	0.14	0.24
5	Baseline	52.55	26.28	26.28	0.26	0.03	0.49
	1	35.87	29.30	6.57	0.19	0.14	0.70
	2	36.39	29.57	6.82	0.19	0.14	0.69
6	Baseline	18.88	9.44	9.44	0.55	0.55	0.56
	1	17.31	8.92	8.39	0.55	0.67	0.68
	2	18.57	9.32	9.26	0.54	0.66	0.66

The average proportion of toxicities observed in the biomarker negative subgroup under the baseline method decreases for Scenario 1 through 5, while that in the biomarker negative group increases. This is for no difference in the number of patients treated between subgroups. This contrasting proportion of DLTs observed in the two subgroups demonstrates that across simulated trials most biomarker negative patients were being underdosed, with an average of only 3*%* experiencing DLTs in Scenario 5. This contrasts with the average of 49*%* of biomarker positive patients treated experienced DLTs in this scenario and hence many were likely overdosed.

It can also be seen that in Scenario 5 under the baseline method, an average of 26.28 patients were treated in the biomarker positive subgroup per trial despite there being no tolerated dose in this subgroup. This is compared with around 7 biomarker positive patients treated under the methods which accounted for a subgroup effect. It is the ability of the methods which account for a potential subgroup effect to stop for safety in one subgroup but continue escalation in the other that leads to this advantage.

The reduced number of patients treated in the biomarker positive subgroup under Methods 1 and 2 in Scenario 5, and the sample sizes observed for both subgroups in Scenario 6, show that the stopping criterion for safety is effective. It had the effect of reducing the overall average sample size from 60 to below 19 when there was no tolerated dose in either subgroup. In that scenario (Scenario 6), all methods were comparable, with around 90*%* of trials correctly identifying that there was no tolerated dose in either subgroup (Table 5). The baseline method was comparable to the alternative in this case because its underlying assumption, that there was no subgroup effect, was correct.

**Table 5:** Number of trials which identified a subgroup effect (0= no subgroup effect, 1= significant subgroup effect, 2= defaulted to subgroup effect after stopping for safety in one subgroup) and proportion of times each dose was recommended by subgroup out of trials giving a recommended dose (based on a frequentist calculation). Grey cells highlight dose‐pairs with probability of causing a DLT in a patient greater than 0.35. The “X” marks the dose with probability of toxicity closest to 0.16

					Recommended Dose													
	Escalation	Significant Subgroup Effect			${𝕝}_{+}=0$							${𝕝}_{+}=1$						
Scenario	Method	0	1	2	0	100	150	180	215	245	260	0	100	150	180	215	245	260
1	Baseline	1000	0	0	0.01	0.01	0.05	0.49	0.36^*X*^	0.07	0.02	0.01	0.01	0.05	0.49	0.36^*X*^	0.07	0.02
	1	0	951	49	0.02	0.02	0.11	0.39	0.33^*X*^	0.08	0.04	0.03	0.02	0.10	0.38	0.33^*X*^	0.09	0.04
	2	666	298	36	0.03	0.01	0.09	0.40	0.36^*X*^	0.09	0.03	0.02	0.01	0.10	0.40	0.36^*X*^	0.08	0.03
2	Baseline	1000	0	0	0.01	0.01	0.11	0.58	0.28^*X*^	0.02	0.00	0.01	0.01	0.11	0.58^*X*^	0.28	0.02	0.00
	1	0	962	38	0.03	0.01	0.11	0.42	0.32^*X*^	0.07	0.04	0.02	0.03	0.25	0.49^*X*^	0.19	0.02	0.00
	2	662	304	34	0.02	0.02	0.11	0.45	0.32^*X*^	0.06	0.03	0.02	0.03	0.20	0.50^*X*^	0.22	0.02	0.01
3	Baseline	1000	0	0	0.00	0.01	0.34	0.59	0.06^*X*^	0.00	0.00	0.00	0.01	0.34^*X*^	0.59	0.06	0.00	0.00
	1	0	945	55	0.02	0.02	0.13	0.36	0.32^*X*^	0.10	0.04	0.04	0.13	0.55^*X*^	0.26	0.01	0.00	0.00
	2	423	511	66	0.03	0.01	0.17	0.41	0.26^*X*^	0.08	0.04	0.05	0.10	0.47^*X*^	0.35	0.03	0.00	0.00
4	Baseline	1000	0	0	0.01	0.30	0.68	0.01	0.00^*X*^	0.00	0.00	0.01	0.30^*X*^	0.68	0.01	0.00	0.00	0.00
	1	0	871	129	0.03	0.02	0.12	0.40	0.32^*X*^	0.08	0.03	0.11	0.76^*X*^	0.13	0.00	0.00	0.00	0.00
	2	73	804	123	0.02	0.04	0.13	0.36	0.34^*X*^	0.09	0.03	0.11	0.74^*X*^	0.15	0.00	0.00	0.00	0.00
5	Baseline	1000	0	0	0.17	0.83	0.00	0.00	0.00^*X*^	0.00	0.00	0.17^*X*^	0.83	0.00	0.00	0.00	0.00	0.00
	1	0	69	931	0.03	0.02	0.11	0.39	0.32^*X*^	0.09	0.04	0.95^*X*^	0.05	0.00	0.00	0.00	0.00	0.00
	2	7	62	931	0.02	0.02	0.11	0.37	0.36^*X*^	0.08	0.04	0.95^*X*^	0.05	0.00	0.00	0.00	0.00	0.00
6	Baseline	1000	0	0	0.89^*X*^	0.10	0.00	0.00	0.00	0.00	0.00	0.89^*X*^	0.10	0.00	0.00	0.00	0.00	0.00
	1	0	183	817	0.89^*X*^	0.10	0.00	0.00	0.00	0.00	0.00	0.91^*X*^	0.09	0.00	0.00	0.00	0.00	0.00
	2	323	0	677	0.90^*X*^	0.10	0.00	0.00	0.00	0.00	0.00	0.90^*X*^	0.10	0.00	0.00	0.00	0.00	0.00

In Scenario 1, the bulk of recommended doses by all methods are split between 180 and 215 mg/m^2^. This is not completely unexpected as the true TD16 for this scenario is 206 mg/m^2^ which falls between the 2 but being slightly closer to 215 mg/m^2^. The true recommended doses, along with the probability of toxicity for all scenarios are given in Table 3. The locations of the recommended doses in Scenario 1 were also similar across all methods. This suggests that when a suitable number of patients are treated in each subgroup (with 30 appearing to be suitable), the recommended dose is identified with a reasonable level of accuracy, even when there is no subgroup effect.

Now, consider the locations of recommended doses from Scenarios 2 to 5 (Table 5). As the subgroup effect increased, the baseline method got progressively worse. This is because, under the baseline method, the assumption is that all observations arise from the same population; the resulting recommended dose is effectively a compromise between the true recommended doses from the two subgroups. The most undesirable outcome from the baseline method arises from Scenario 5 where the true recommended dose in the biomarker negative subgroup was 215 mg/m^2^ and there was no tolerated dose in the biomarker positive subgroup. In 17*%* of trials, the baseline method stopped for safety in both subgroups, and in the remaining trials it identified the recommended dose for the entire population as 100 mg/m^2^. This means that 83*%* of the time a dose which had “true” DLT rate 0.02 (expected to be inefficacious) and 0.42 (undesirably toxic) in the two subgroups was recommended for further testing.

Method 1, which considers a potential subgroup effect throughout escalation and in dose recommendation, performed much better than the baseline. This suggests that 30 patients, with the levels of variability observed here, are suitable to identify a recommended dose in a homogeneous population with reasonable accuracy. As previously discussed, ideally we would like some idea of whether a subgroup effect was in fact observed.

Method 2 was designed to avoid this problem and did so successfully. Only small differences in recommended dose locations were seen between the baseline method and Method 2 in Scenario 1, with a conclusion of no subgroup effect under Method 2 66.6*%* of the time. In the presence of a medium subgroup effect (as in Scenarios 3 and 4), the spike and slab priors were effective in identifying a subgroup effect. The proportion of times a subgroup effect was correctly identified in Scenarios 3 and 4 was 57.7*%* and 92.7*%*, respectively. Although the recommended dose locations from Method 2 were similar to those from Method 1, Method 2 has the advantage of providing exploratory information concerning the presence of a subgroup effect. In addition to the simulation results presented in this manuscript, Method 2 was run with a maximum of 120 patients per subgroup. From these results, we were able to conclude that given a suitable number of patients, this method provides good estimation of the recommended dose in each subgroup.

#### 3.2.1 Allowing early stopping for accuracy

Although a total of 30 patients (or more) in each subgroup is desirable, it is not always feasible. Along with the stopping rules which were used in the previous simulations (for safety in a subgroup or having treated the maximum number of patients in each subgroup), we now include one for accuracy. That is, the trial can stop for accuracy in a subgroup if a minimum of 5 patients have been treated at the dose advised for administration to the next cohort of patients and the 95*%* credible interval around the estimate of that dose is less than 5 (as used in Whitehead et al^39^). We compare the impact of this stopping rule on Methods 1 and 2. The baseline design is not considered here because we have already confirmed that it is not suitable when a subgroup effect is present. In a homogeneous population, the effect of stopping rules is similar to that seen in one subgroup for Method 1.

Introducing the stopping rule for accuracy was effective in reducing the sample size of the trial; this can be seen from the operating characteristics of the methods presented in Table 6. In Scenarios 1 to 4, where there was a tolerated dose in each subgroup, the average number of patients in the trial was between 45 and 51 in both methods. Even based on these reduced sample sizes, the locations of the recommended doses were still compacted around the true recommended dose; this can be seen in Table 7 for both methods. Table 8 shows the reasons that trials stopped.

**Table 6:** Average number of patients treated per trial in total and in each subgroup, average proportion of toxicities observed per trial in total and in each subgroup, in simulations which allow early stopping for accuracy

	Escalation	Average Number Patients			Average Proportion Toxicities		
Scenario	Method	Overall	${𝕝}_{+}=0$	${𝕝}_{+}=1$	Overall	${𝕝}_{+}=0$	${𝕝}_{+}=1$
1	1	48.80	24.12	24.68	0.12	0.14	0.14
	2	47.36	23.70	23.66	0.11	0.13	0.12
2	1	48.22	24.39	23.83	0.13	0.14	0.16
	2	47.90	23.31	24.59	0.12	0.13	0.15
3	1	49.29	24.99	24.29	0.14	0.13	0.18
	2	47.77	22.01	25.77	0.13	0.11	0.18
4	1	50.84	24.51	26.33	0.15	0.14	0.23
	2	45.40	18.94	26.46	0.15	0.12	0.26
5	1	32.55	25.58	6.97	0.19	0.14	0.68
	2	26.87	20.03	6.84	0.20	0.12	0.71
6	1	19.19	9.45	9.74	0.53	0.65	0.66
	2	18.80	9.13	9.66	0.53	0.67	0.65

**Table 7:** Proportion of times each dose was recommended by subgroup out of trials giving a recommended dose (based on a frequentist calculation), in simulations which allow early stopping for accuracy. Grey cells highlight dose‐pairs with probability of causing a DLT in a patient greater than 0.35. The “X” marks the dose with probability of toxicity closest to 0.16

		Recommended Dose													
	Escalation	${𝕝}_{+}=0$							${𝕝}_{+}=1$						
Scenario	Method	0	100	150	180	215	245	260	0	100	150	180	215	245	260
1	1	0.04	0.02	0.13	0.33	0.32^*X*^	0.08	0.09	0.03	0.01	0.14	0.35	0.34^*X*^	0.06	0.08
	2	0.03	0.01	0.11	0.43	0.26^*X*^	0.10	0.06	0.02	0.02	0.10	0.42	0.24^*X*^	0.12	0.07
2	1	0.03	0.02	0.14	0.34	0.32^*X*^	0.06	0.09	0.03	0.03	0.24	0.43^*X*^	0.23	0.02	0.02
	2	0.03	0.02	0.12	0.44	0.22^*X*^	0.10	0.07	0.03	0.03	0.23	0.48^*X*^	0.17	0.05	0.02
3	1	0.02	0.03	0.12	0.35	0.32^*X*^	0.08	0.09	0.04	0.12	0.46^*X*^	0.32	0.05	0.00	0.00
	2	0.02	0.02	0.16	0.41	0.20^*X*^	0.11	0.07	0.05	0.10	0.45^*X*^	0.36	0.04	0.01	0.00
4	1	0.03	0.01	0.13	0.35	0.31^*X*^	0.07	0.09	0.11	0.74^*X*^	0.14	0.00	0.00	0.00	0.00
	2	0.03	0.02	0.11	0.41	0.20^*X*^	0.13	0.10	0.12	0.74^*X*^	0.14	0.00	0.00	0.00	0.00
5	1	0.02	0.02	0.12	0.38	0.29^*X*^	0.09	0.07	0.94^*X*^	0.06	0.00	0.00	0.00	0.00	0.00
	2	0.02	0.02	0.11	0.43	0.25^*X*^	0.11	0.07	0.93^*X*^	0.07	0.00	0.00	0.00	0.00	0.00
6	1	0.89^*X*^	0.11	0.00	0.00	0.00	0.00	0.00	0.88^*X*^	0.12	0.00	0.00	0.00	0.00	0.00
	2	0.90^*X*^	0.10	0.00	0.00	0.00	0.00	0.00	0.89^*X*^	0.11	0.00	0.00	0.00	0.00	0.00

**Table 8:** Proportion of trials which stopped for safety, having treated the maximum number of patients and for accuracy in each subgroup

		Reason Trial Stopped					
	Escalation	${𝕝}_{+}=0$			${𝕝}_{+}=1$		
Scenario	Method	Safety	Max	Accuracy	Safety	Max	Accuracy
1	1	0.03	0.50	0.49	0.02	0.55	0.45
	2	0.02	0.54	0.46	0.01	0.54	0.45
2	1	0.02	0.53	0.47	0.03	0.50	0.49
	2	0.02	0.52	0.47	0.03	0.62	0.36
3	1	0.01	0.56	0.45	0.04	0.57	0.40
	2	0.01	0.41	0.59	0.04	0.73	0.23
4	1	0.02	0.52	0.47	0.11	0.84	0.05
	2	0.02	0.23	0.77	0.12	0.87	0.01
5	1	0.01	0.56	0.46	0.92	0.08	0.00
	2	0.01	0.24	0.77	0.92	0.09	0.00
6	1	0.85	0.15	0.00	0.83	0.16	0.00
	2	0.87	0.13	0.00	0.85	0.15	0.00

We see that in Scenario 1, under both methods, 45% to 49*%* of trials stopped early for accuracy in both subgroups. In Method 1, for Scenarios 2 to 5, the proportion of trials which stopped early for accuracy was consistently around these values when there was a tolerated dose in the subgroup. In Method 2, the proportion of trials which stopped for accuracy in the biomarker negative subgroup increased as the true subgroup effect increased, while decreasing in the biomarker positive subgroup. The reason for this large discrepancy was model selection identifying the presence of a subgroup effect; it was therefore better able to estimate the dose‐toxicity curve in the biomarker negative subgroup due to the spread of data. On the other hand, the high uncertainty surrounding the estimation of the dose‐toxicity curve in the biomarker positive subgroup, caused by a lack of data at higher doses, leads to a reduced number of trials stopping for accuracy as the subgroup effect increased.

As expected, the stopping rule for accuracy did not come in to play in a subgroup in which there was no tolerated dose (as in the biomarker positive subgroup in Scenario 5 and both subgroups in Scenario 6). This was down to the stopping rule for safety being met.

## 4 DISCUSSION

In this paper, we demonstrated methods which extend a traditional dose‐toxicity model used in dose‐escalation to account for a potential subgroup effect by including terms for subgroup membership. In doing so, the assumption of a homogeneous trial population is removed, reducing the risk of a missed or masked treatment effect due to variability between subgroups of the population. The dose‐escalation methods presented which account for a potential subgroup effect follow a similar procedure to the standard Bayesian model‐based design to which they were compared. In this way, after the initial set‐up of the trial, they should be no more difficult to employ.

Simulation results showed that accounting for subgroup membership in dose‐escalation can increase the safety of escalation. Importantly, Methods 1 and 2 had the ability to stop early for safety in a subgroup if there was no tolerated dose, reducing the number of overdoses recommended for use in future trials. Simulation results showed that the novel method, which used spike and slab priors on the terms for subgroup membership (presented as Method 2), was reasonably good at identifying the presence of an underlying subgroup. The recommended dose locations from Method 2 were similar to those from Method 1 but with the advantage of providing exploratory information concerning the presence of a subgroup effect. Also, when there was no identifiable subgroup effect, escalation and identification of the recommended dose makes better use of available data than Method 1.

The methods were initially compared with a total of 30 patients available for treatment in each subgroup. Although such a sample size would be desirable, it is not always feasible. The use of a stopping rule for accuracy demonstrated that an overall sample size of 45 to 50 was suitable for Methods 1 and 2 to identify a recommended dose with a relatively small loss in accuracy under the scenarios investigated.

As with standard Bayesian model‐based designs, the proposed method is flexible and practical since available doses and cohort sizes, among other design factors, can be altered throughout the trial. The optimal setting with cohorts of size 2, consisting of one biomarker positive and one biomarker negative patient (unless one subgroup had stopped for safety), was considered. This could be altered but the more unevenly distributed the patients are between subgroups, the worse the model selection algorithm in Method 2 will perform. The proposed methods can allow for different values of *θ* to be used in each subgroup, if required. In practice it is also still possible for the clinical team to over‐ride the model decision based on any available data.

Simulations were based on the scenario that patients could be reliably divided into 2 disjoint subgroups. For some biomarkers, such as pretreatment, this will be the case. There may be other biomarkers of interest, such as those defined based on assay results, which have lower accuracy. Accuracy of the biomarker decreases the performance of the proposed methods will get closer to the method which does not account for subgroup membership.

We specified a Dirac delta function for the ‘spike’ component of the prior on the terms for subgroup membership. Alternative choices include use of a normal distribution with large mass at zero and a double exponential model (or Lasso, see Tibshirani^40^ for details). Although a mixture of normal distributions results in a continuous prior, it is one which is not sparsity inducing. As a result, a straight‐forward decision concerning whether a term should be included in the model cannot be made. Bernardo et al^41^ compare a range of prior settings, including those mentioned, and obtain no clear conclusion over the “better” sparsity inducing prior.

A method related to Bayesian variable selection is Bayesian model averaging.^42^ Although such methods would be feasible with the small number of parameters in our model, we wish to obtain a clear decision over whether the terms for subgroup membership should be included in the model. For this reason, we choose to use variable selection. Bayes factor or penalised regression are other alternative methods which reduce the challenges involved in specifying the spike and slab prior and related inclusion probabilities. These methods may be of interest for future investigation but were not included in this manuscript as they are not fully Bayesian and, hence, do not readily allow incorporation of prior information.

The methods discussed in this manuscript only have the potential to highlight subgroup effects between the two predefined subgroups of the population. It could be beneficial to extend this to the ordinal setting (similar to that of Tighioutart et al^43^). However, the sample size in dose‐escalation trials is usually too small to consider identification of a subgroup effect, with suitable power, within the trial. Rogatko et al^44^ propose extending the search for the optimal dose, and consideration of a subgroup effect, beyond dose‐escalation. This can also help account for population changes and longer‐term endpoints in the identification of an optimal dose.

## Figures and Tables

**Figure A1:**
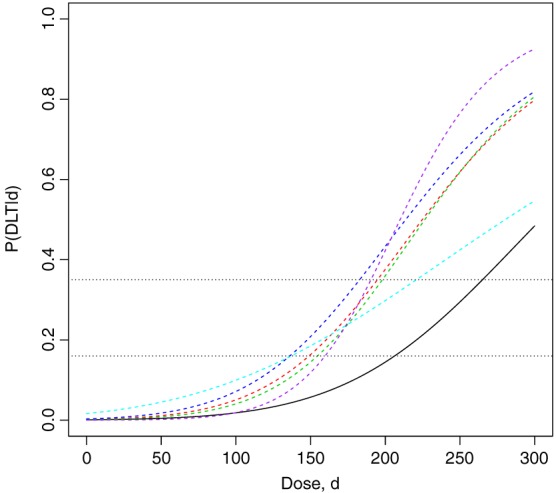
The dose‐toxicity curves used to generate data in additional Scenarios 7 to 11. Horizontal lines are references at P(DLT|d)=0.16 and 0.35. The solid black curve on each plot represents that of the biomarker negative subgroup in all scenarios. The dose‐toxicity curves for the biomarker positive group in these scenarios are shown for Scenarios 7 to 11 by the dashed red, green, dark blue, light blue and purple curves, respectively

## APPENDIX A

### PRIOR SPECIFICATION

We chose to specify the prior to control the operating characteristics of the trial. This required investigation of the likely escalation patterns of a range of prior settings. We specified no prior subgroup effect (to aid comparison of the methods) and weighted the prior data to 1/10th of the planned trial size. So, in selecting a prior we investigated priors consisting of 3 patients worth of data under dose‐escalation Method 1 in one subgroup.

In order to get a start dose of 100 mg/m^2^, this is selected as the lower of the prior doses with a prior probability of DLT equal to 0.16, the target toxicity level. The higher prior dose, the prior proportion of toxicities at each dose and the weighting of patients at each dose were then altered in the investigated prior settings. These are given in Table A1.

**Table A1:** Prior settings tested given in terms of the prior proportion of DLTs observed at each prior dose and, in brackets, the number of prior patients considered at that dose out of the total of 3 patients. The ‘*’ indicates the prior setting used in the simulation study

Prior	Prior Pseudo‐data DLT Outcomes by Dose (mg/m^2^)					
setting	100	150	180	215	245	260
1	1/6 (1.5)	‐	‐	1/3 (1.5)	‐	‐
2	1/6 (1.5)	‐	‐	‐	‐	1/3 (1.5)
3	1/6 (1.5)	‐	‐	‐	‐	1/2 (1.5)
4	1/6 (1.5)	‐	‐	‐	‐	2/3 (1.5)
5	1/6 (2)	‐	‐	‐	‐	1/3 (1)
6^∗^	1/6 (2)	‐	‐	‐	‐	1/2 (1)
7	1/6 (2)	‐	‐	‐	‐	2/3 (1)
8	1/6 (1)	‐	‐	‐	‐	1/3 (2)
9	1/6 (1)	‐	‐	‐	‐	1/2 (2)

Under the scenario in which no DLTs were observed during dose‐escalation, prior settings 1 and 2 led to dose levels being skipped. Prior settings 5, 8, and 9 were also felt to escalate too rapidly, requiring observation of only one patient at some doses before escalating. Further scenarios were investigated in which a DLT was observed early on in the trial. Under prior settings 3 and 4, observation of a single DLT led to de‐escalation by two dose levels which appeared overly cautious. Prior settings 6 and 7 de‐escalated by only one dose level with observation of a single DLT. Prior setting 6 was selected for use in simulations over setting 7 because under setting 7, re‐escalation after observation of a DLT was considered potentially too slow given the small number of patients available in the trial.

## APPENDIX B

### SENSITIVITY ANALYSIS

The purpose of the additional simulations presented here was to investigate the sensitivity of the methods to different parameter values in the data generating dose‐toxicity model. The same parameter values used to generate data for both subgroups in Scenario 1 was used for the biomarker negative subgroup, resulting in a true recommended dose of 215 mg/m^2^ in this subgroup in all cases. For the biomarker positive subgroup, the values of *β*_2_ and *β*_3_ were altered to create different scenarios in a way that resulted in a true recommended dose of 150 mg/m^2^ in each case. The resulting dose‐toxicity curves are shown in Figure A1. The corresponding true probability of DLT at each available dose is given in Table B1.

**Table B1:** Parameter values and simulated probability of DLT at each dose (in mg/m^2^) and simulation scenario in additional simulations, given for the biomarker positive subgroup. Grey cells highlight dose‐pairs with probability of causing a DLT in a patient greater than 0.35. The “X” marks the dose with probability of toxicity closest to 0.16, in cases where there is a tolerated dose

	Parameter value				P(DLT $|d,{𝕝}_{+}=1$)					
Scenario	*β*_0_	*β*_1_	*β*_2_	*β*_3_	100	150	180	215	245	260
1	−7.10	7.68	0.00	0.00	0.02	0.06	0.10	0.18^*X*^	0.28	0.33
7	−7.10	7.68	0.75	0.75	0.05	0.16^*X*^	0.28	0.45	0.60	0.66
8	−7.10	7.68	0.30	1.30	0.04	0.15^*X*^	0.26	0.44	0.59	0.66
9	−7.10	7.68	1.30	0.30	0.07	0.21^*X*^	0.34	0.51	0.64	0.70
10	−7.10	7.68	3.00	−3.00	0.10	0.19^*X*^	0.25	0.34	0.41	0.45
11	−7.10	7.68	−2.00	5.00	0.02	0.12^*X*^	0.28	0.54	0.74	0.81

From the locations of the recommended doses for these additional scenarios, which are presented in Table B2, we can confirm that we have run a suitable number of simulations to be relatively certain in our conclusions drawn, for the given setting. This is seen from the consistency in the outcomes of the biomarker negative subgroup. The rest of this discussion is focussed on operating characteristics in the biomarker positive subgroup.

**Table B2:** Number of trials which identified a subgroup effect (0= no subgroup effect, 1= significant subgroup effect, 2= defaulted to subgroup effect after stopping for safety in one subgroup) and proportion of times each dose was recommended by subgroup out of trials giving a recommended dose (based on a frequentist calculation), for Scenarios 7‐11. Grey cells highlight dose‐pairs with probability of causing a DLT in a patient greater than 0.35. The “X” marks the dose with probability of toxicity closest to 0.16

					Recommended Dose													
	Escalation	Significant Subgroup Effect			${𝕝}_{+}=0$							${𝕝}_{+}=1$						
Scenario	Method	0	1	2	0	100	150	180	215	245	260	0	100	150	180	215	245	260
7	Baseline	1000	0	0	0.01	0.02	0.48	0.45	0.04^*X*^	0.00	0.00	0.01	0.02	0.48^*X*^	0.45	0.04	0.00	0.00
	1	0	936	64	0.02	0.02	0.13	0.37	0.33^*X*^	0.09	0.04	0.05	0.21	0.58^*X*^	0.15	0.01	0.00	0.00
	2	364	567	69	0.02	0.02	0.21	0.37	0.29^*X*^	0.06	0.03	0.06	0.20	0.53^*X*^	0.20	0.02	0.00	0.00
8	Baseline	1000	0	0	0.01	0.02	0.42	0.52	0.04^*X*^	0.00	0.00	0.01	0.02	0.42^*X*^	0.52	0.04	0.00	0.00
	1	0	928	72	0.03	0.01	0.11	0.41	0.33^*X*^	0.07	0.04	0.05	0.16	0.57^*X*^	0.21	0.01	0.00	0.00
	2	399	540	61	0.02	0.02	0.19	0.39	0.28^*X*^	0.07	0.03	0.04	0.15	0.51^*X*^	0.27	0.32	0.00	0.00
9	Baseline	1000	0	0	0.00	0.07	0.65	0.27	0.01^*X*^	0.00	0.00	0.00	0.07	0.65^*X*^	0.27	0.01	0.00	0.00
	1	0	896	104	0.02	0.01	0.13	0.41	0.32^*X*^	0.07	0.04	0.10	0.40	0.45^*X*^	0.06	0.00	0.00	0.00
	2	268	636	96	0.02	0.03	0.21	0.36	0.28^*X*^	0.07	0.04	0.09	0.33	0.46^*X*^	0.11	0.01	0.00	0.00
10	Baseline	1000	0	0	0.01	0.08	0.42	0.41	0.07^*X*^	0.01	0.00	0.01	0.08	0.42^*X*^	0.41	0.07	0.01	0.00
	1	0	860	140	0.02	0.01	0.14	0.40	0.31^*X*^	0.08	0.04	0.14	0.31	0.36^*X*^	0.14	0.03	0.00	0.00
	2	336	535	129	0.03	0.03	0.18	0.37	0.29^*X*^	0.07	0.04	0.12	0.31	0.33^*X*^	0.19	0.04	0.00	0.00
11	Baseline	1000	0	0	0.00	0.00	0.38	0.60	0.01^*X*^	0.00	0.00	0.00	0.00	0.38^*X*^	0.60	0.01	0.00	0.00
	1	0	972	28	0.02	0.02	0.13	0.40	0.32^*X*^	0.08	0.04	0.01	0.08	0.65^*X*^	0.26	0.00	0.00	0.00
	2	406	559	35	0.03	0.01	0.19	0.37	0.27^*X*^	0.09	0.04	0.02	0.08	0.57^*X*^	0.31	0.02	0.00	0.00

It is difficult to make any firm conclusions concerning the effect of each of the parameters on the methods but it is clear that the overall comparisons between the methods which we have already made stand in all cases. Despite the different parameter values used to generate data in Scenarios 7 and 8, the resulting dose‐toxicity curves were fairly similar over the dose range of interest. This is likely to be the reason that the operating characteristics of these scenarios are similar. Although the dose‐toxicity curve for Scenario 9 is not greatly dissimilar to those of Scenarios 7 and 8, there appeared to be an increased chance of stopping early. This could be due to the value of *β*_2_ being greater than *β*_3_ because this observation was more evident in Scenario 10 which had an even larger difference in parameter values. Scenario 11 resulted in a dose‐toxicity curve with low toxicity at low doses but then a sharp increase. The average proportion of toxicities observed in the trial were therefore decreased and fewer trials stopped for safety.

### B.2 Investigating inclusion probabilities

We investigated the effect of the prior inclusion probability of *β*_2_ and *β*_3_ and also the boundary on the inclusion probability for inclusion of terms in the fitted model. The combinations investigated are given in Table B3. The prior pseudo‐data used in simulations was the same for both subgroups and, hence, it may be counter‐intuitive to place a high prior probability of inclusion on *β*_2_ and *β*_3_. Specification of these parameters will depend on available prior data and operating characteristics of the design which should be investigated through simulation prior to implementation.

**Table B3:** Combinations of prior inclusion probability and boundary for inclusion of terms included in the model investigated in Method 2

Method	Prior	Prior Inclusion Probability		Boundary for Inclusion
	Setting	*β*_2_	*β*_3_	of Term in Model
3	a	0.3	0.3	0.25
	b	0.3	0.3	0.35
	c	0.5	0.5	0.25
	d	0.5	0.5	0.35
	e	0.7	0.7	0.25
	f	0.7	0.7	0.35

As expected, the average number of patients and proportion of DLTs were very similar in each of the inclusion probability settings. This confirms the safety criterion on escalation is effective and that, in general, escalation is targeting suitable doses. The effect of the inclusion probability parameters on the model choice also agreed with expectations. This can be seen from the number of trials which declared a significant subgroup effect in escalation, as shown in Table B4 for prior settings 1 and 3. Increasing the prior inclusion probability of the parameters led to the terms for subgroup membership being included in the model more often. Increasing the bound for inclusion of a term in the model led to a decrease in how often the terms for subgroup membership were considered in the model, and hence how many trials concluded that a significant subgroup effect was present. Simulations were also run using boundary for inclusion of 0.50 with performance consistent with observations already made.

**Table B4:** Number of trials which identify a subgroup effect (0= no subgroup effect, 1= significant subgroup effect, 2= defaulted to subgroup effect after stopping for safety in one subgroup) and proportion of times each dose was recommended by subgroup out of trials giving a recommended dose (based on a frequentist calculation) in Method 2 a range of inclusion probability settings. Grey cells highlight dose‐pairs with probability of causing a DLT in a patient greater than 0.35. The “X” marks the dose with probability of toxicity closest to 0.16

					Recommended Dose													
	PIP	Significant Subgroup Effect			${𝕝}_{+}=0$							${𝕝}_{+}=1$						
Scenario	Setting	0	1	2	0	100	150	180	215	245	260	0	100	150	180	215	245	260
1	a	889	78	33	0.02	0.01	0.04	0.44	0.44^*X*^	0.04	0.01	0.01	0.00	0.05	0.44	0.44^*X*^	0.04	0.01
	b	906	46	48	0.02	0.01	0.05	0.48	0.39^*X*^	0.05	0.01	0.03	0.00	0.04	0.48	0.38^*X*^	0.06	0.01
	c	732	244	24	0.02	0.01	0.06	0.43	0.41^*X*^	0.07	0.02	0.01	0.01	0.08	0.42	0.40^*X*^	0.06	0.02
	d	827	140	33	0.02	0.01	0.06	0.45	0.39^*X*^	0.07	0.02	0.02	0.01	0.05	0.44	0.40^*X*^	0.07	0.02
	e	287	668	45	0.03	0.01	0.10	0.43	0.32^*X*^	0.07	0.03	0.03	0.01	0.09	0.41	0.37^*X*^	0.07	0.02
	f	535	431	34	0.02	0.01	0.07	0.41	0.29^*X*^	0.07	0.03	0.02	0.01	0.08	0.42	0.38^*X*^	0.07	0.02
3	a	636	307	57	0.02	0.01	0.20	0.51	0.20^*X*^	0.06	0.01	0.04	0.06	0.41^*X*^	0.46	0.03	0.00	0.00
	b	743	207	50	0.02	0.01	0.22	0.53	0.17^*X*^	0.04	0.01	0.04	0.05	0.37^*X*^	0.50	0.04	0.00	0.00
	c	429	514	57	0.02	0.00	0.14	0.44	0.30^*X*^	0.07	0.02	0.04	0.08	0.50^*X*^	0.35	0.04	0.00	0.00
	d	560	386	54	0.02	0.01	0.18	0.48	0.23^*X*^	0.05	0.02	0.04	0.08	0.44^*X*^	0.41	0.03	0.00	0.00
	e	133	819	48	0.02	0.01	0.10	0.42	0.35^*X*^	0.08	0.03	0.03	0.09	0.57^*X*^	0.30	0.01	0.00	0.00
	f	261	677	62	0.01	0.01	0.12	0.43	0.33^*X*^	0.08	0.02	0.05	0.08	0.57^*X*^	0.29	0.01	0.00	0.00
